# Hyperphosphatemic Familial Tumoral Calcinosis With *Galnt3* Mutation: Transient Response to Anti‐Interleukin‐1 Treatments

**DOI:** 10.1002/jbm4.10185

**Published:** 2019-03-06

**Authors:** Astrid Dauchez, Camille Souffir, Pierre Quartier, Geneviève Baujat, Karine Briot, Christian Roux

**Affiliations:** ^1^ Rheumatology Department Cochin Hospital Assistance Publique − Hôpitaux de Paris Paris France; ^2^ Paris Descartes University Paris France; ^3^ Paediatric Immunology − Haematology and Rheumatology Department Necker Hospital Assistance Publique − Hôpitaux de Paris Paris France; ^4^ IMAGINE Institute Paris France; ^5^ French National Reference Centre for Inflammatory Rheumatism and Autoimmune Systemic Disease in Children (RAISE); ^6^ National Reference Center for Genetic Bone Diseases

**Keywords:** HYPERPHOSPHATEMIC FAMILIAL TUMORAL CALCINOSIS, ANTI‐INTERLEUKINE‐1 THERAPIES, ANAKINRA, CANAKINUMAB

## Abstract

Hyperphosphatemic familial tumoral calcinosis (HFTC) is a rare autosomal recessive disease caused by mutations in genes involved in phosphate homeostasis and characterized by high serum phosphate concentration and occurrence of ectopic calcifications. Management of the disease includes lowering of phosphate concentration and, when clinically necessary, debulking surgery of calcifications. In addition, high inflammatory disease flares can occur. Our case is about a patient with *GALNT3* mutation and several localizations of refractory calcinosis. Assuming HFTC acts like an auto‐inflammatory syndrome, we report the effect of anti‐interleukine‐1 therapies on the evolution of the disease. Anakinra (100 mg, then 200 mg subcutaneous daily) and canakinumab (300 mg every 4 weeks) were sequentially given to the patient. Anti‐IL‐1 therapy was effective in controlling inflammatory flares; however, it did not prevent extension of calcinosis. © 2019 The Authors. *JBMR Plus* published by Wiley Periodicals, Inc. on behalf of American Society for Bone and Mineral Research.

## Introduction

Hyperphosphatemic familial tumoral calcinosis (HFTC) is a rare autosomal recessive disease, characterized by high serum phosphate concentration and occurrence of ectopic calcifications. It is caused by mutations in genes encoding for phosphate homeostasis: fibroblast growth factor 23 (FGF23),^1^ N‐acetylgalactosaminyltransferase 3 (GALNT3),^2^ or Klotho.^3^ They result in loss of function or resistance to FGF23, which controls the phosphate balance.^4^, ^5^ Thus, HFTC is characterized by high serum phosphate, normal serum calcium and parathyroid hormone, and normal or high 1,25 dihydroxyvitamin D3 (1,25 D).

Management of calcinosis includes surgical treatment in patients with pain and/or functional impairment, but the recurrence rate is high.^6^ Hyperphosphatemia is managed by dietary phosphate restriction, phosphate chelators such as sevelamer,^7^ nicotamide^8^ or treatments increasing phosphate renal excretion such as acetazolamide,^7^, ^9^ and calcitonin.^9^, ^10^ In addition, flare, characterized by severe pain, fever and local and systemic inflammatory reactions can occur. It is presumed that these flares are related to interleukin‐1 (IL‐1) production by macrophages within calcifications.

We report here a case of a patient with severe inflammatory flares of the disease and the effect of anti‐IL‐1 treatments, assuming HFTC acts like an auto‐inflammatory syndrome.

## Description of the Case

**Figure 1:**
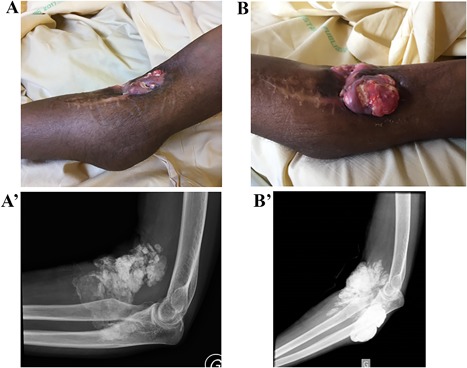
Calcifications surrounding the left elbow. Clinical aspect (with ulcer and visibility of the calcification) and radiographs showing the changes between December 2017 (*A, A’*) and March 2018 (*B, B’*).

The patient, a 20‐year‐old woman, was followed since she was 4 years old for a tumoral calcinosis with a homozygous single‐nucleotide deletion of C in exon 3 of the *GALNT3* gene (c.677delC). Her medical history in infancy was described in a previous study in 2010 (patient 2).^11^ Her parents were first‐degree cousins, and she has no relative known to be affected with the disease. The diagnosis was established during the exploration of tumoral calcinosis that developed around the left elbow and later in several other localizations. The serum phosphate was high, superior to 2.00 mmol/L (normal range: 0.80 to 1.40 mmol/L), while serum calcium level, 1,25 dihydroxyvitamin D3, and PTH were normal. Concentration of C‐terminal FGF23 fragments was elevated with concomitant low intact FGF23 (respectively 563 RU/mL and 11 pg/mL, for normal ranges respectively <150 RU/mL and <71 pg/mL). Some of the lesions were extracted by surgery, but on the left elbow, the calcinosis could only be partly extracted and recurred despite nine surgery procedures between 2004 and 2009. At that time, local fibrosis surrounding vessels and nerves prevented any new surgery. Local aspect is shown on Fig. 1 *A*. During almost each flare occurrence, mild fever was present and white cell counts, erythrocyte sedimentation rate (ESR), and C‐reactive protein (CRP) were increased. The patient was modestly compliant with a low phosphate diet. After the age of 10 years, she was treated with long‐term sevelamer, increased step by step until 6 tablets of 800 mg a day, but she could not tolerate higher doses and other phosphate‐lowering therapies were not attempted. From 2009 to 2014, she also received diltiazem, which seemed useful with faster extrusion of calcinosis masses and faster skin healing. However, it was stopped because of side effects. Nonsteroidal anti‐inflammatory drugs, methotrexate, and the soluble TNF‐receptor etanercept only resulted in mild improvement of pain and fatigue.

**Figure 2:**
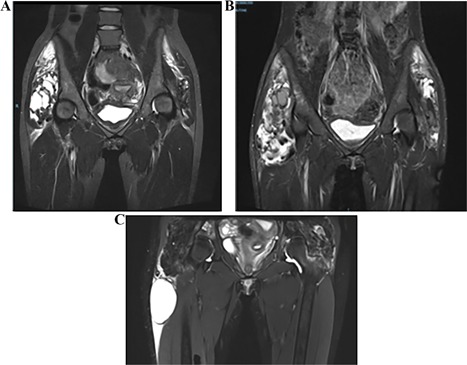
MRI comparison of the hips. (*A*) March 2016: right mass 106 × 121 × 65 mm, left mass 82 × 95 × 60 mm. (*B*) March 2017: right mass 170 × 121 × 65 mm, left mass 112 × 119 × 67 mm. (*C*) March 2018: calcic masses stable; occurrence of milky cyst in right thigh.

**Figure 3:**
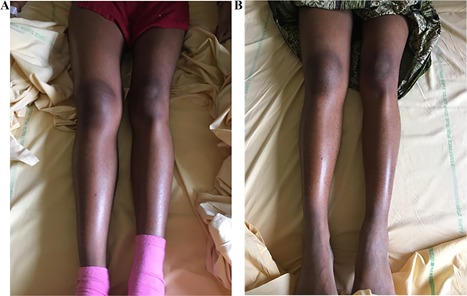
Clinical aspect of the right thigh and the evolution of its diameter. (*A*) Beginning of the flare in December 2017: proximal 58.5 cm, distal 38.5 cm. (*B*) Day 2 after canakinumab initiation: proximal 50 cm, distal 36 cm.

**Figure 4:**
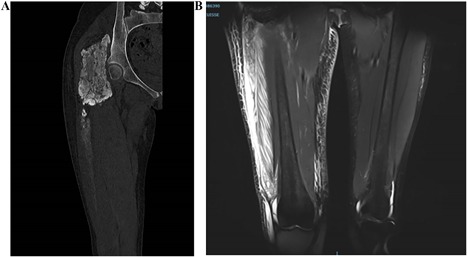
Imaging of the disease flare of December 2017. (*A*) CT scan, bone window: calcic mass in gluteal muscle, erosion of iliac bone for 160 mm with bone condensation, disintegration at the lower pole, extending on more than 200 mm with liquid collection under quadricipital superficial aponeurosis. (*B*) MRI in STIR sequence: collection 300 × 75 × 20 mm with heterogenic content in high STIR signal, with compounds of low STIR signal corresponding with calcic liquid. Muscle swelling; no necrosis.

In 2016, calcinosis around both hips were discovered by palpation of deep masses, confirmed by magnetic resonance imaging (MRI) (Fig. 2 *A*) but causing no pain. In March 2017, the patient became bedridden because of severe pain of right hip and a high inflammatory disease flare: fever, sweating, and CRP over 250 mg/L. MRI showed swelling and extension of the hip calcifications (Fig. 2 *B*). The patient was treated with the recombinant IL‐1 receptor antagonist anakinra 100 mg daily subcutaneously. Pain dramatically decreased at day 1, allowing normal walking at day 3, then CRP was reduced at day 5 (Table 1) and normalized over 3 weeks. During the following weeks, spacing out the injections was not possible as pain quickly reappeared. With daily injections, neither pain nor inflammation occurred for 9 months. In December 2017, while receiving this treatment, a new flare occurred with severe pain, increase in volume of the right thigh again, (shown in Fig. 3 *A*), and increase of CRP over 100 mg/L. The CT scan showed, in addition to the known calcic mass, a calcic spindle along the muscle of the thigh extending for more than 20 cm. The MRI showed an image of “calcic bursitis” along the muscle with huge local swelling but no sign of infection, necrosis, or hematoma (Fig. 4). Considering the intensity of inflammation, three methylprednisolone intravenous injections of 500 mg were used to control it. They were effective on CRP (decreased from 109.6 mg/L to 3.7 mg/L [Table 1]) and on the diameter of the thigh (the proximal diameter decreased from 58.5 cm to 50 cm and the distal diameter from 39.5 cm to 36 cm [Fig. 3 *B*]). Pain was still refractory despite the use of opioids, and a double of anakinra was tested for 12 days, which was ineffective. Thus, we used canakinumab, an IL‐1β antibody 300 mg every 4 weeks, subcutaneously. Over 2 days, the pain decreased, allowing discontinuation of analgesics and the recovery of normal walk. CRP remained low (but not normal) and stable (Table 1).

**Table 1:** Complete Blood Count (Leukocytes, Hemoglobin, and Platelets), CRP, ESR, Serum Calcium, and Phosphate Level Values From March 2017 to March 2018

	3/9/17, hips flare	3/15/17, day 5 anakinra	4/17, month 1 anakinra	5/17, month 2 anakinra	7/1/17, month 4 anakinra	12/1/17, right thigh flare
Leukocytes (4–10 G/L)^a^	7.6	NA	NA	NA	NA	9.27
Hemoglobin (12–17 mg/dL)^a^	7.7	NA	11.7	NA	11.2	10.9
Platelets (150–450 G/L)^a^	586	NA	345	NA	NA	355
CRP (<5 mg/L)^a^	254.3	36.6	3	3	9	109.6
ESR (1–20 mm)^a^	NA	124	36	29	NA	42
Serum calcium (2.25–2.60 mmol/L)^a^	2.6	2.47	2.45	NA	NA	2.36
Serum phosphate (0.80–1.40 mmol/L)^a^	2.14	2.16	2.1	NA	NA	1.75

	12/5/17, after steroid treatment	12/23/17, day 2 canakinumab	1/22/18, month 1 canakinumab	2/20/18, month 2 canakinumab	3/19/18, month 3 canakinumab
Leukocytes (4–10 G/L)^a^	7.96	5.11	7.67	7.15	6.54
Hemoglobin (12–17 mg/dL)^a^	10.3	9.9	10.9	10.5	10.1
Platelets (150–450 G/L)^a^	404	267	314	318	334
CRP (<5 mg/L)^a^	16.4	10.4	3.7	13.8	9.8
ESR (1–20 mm)^a^	NA	NA	23	19	34
Serum calcium (2.25–2.60 mmol/L)^a^	NA	2.47	2.41	2.4	2.41
Serum phosphate (0.80–1.40 mmol/L)^a^	NA	2.59	2.05	2.09	2.56

CRP = C‐reactive protein; ESR = erythrocyte sedimentation rate; NA = not available.

^a^ Normal range and measuring unit.

After two canakinumab injections, there was no more pain and no clinical increase of volume of the right thigh, but the CRP was still elevated at 13.8 mg/L. However, after the third canakinumab injection, the patient experienced new pain in the left thigh, a growing of the left elbow calcic masses, but a stable CRP level at 9.8 mg/L. An emergency CT scan eliminated new calcinosis of the left thigh and the planned control MRI of the right thigh showed a modification of calcic cysts but no extension or inflammatory sign (Fig. 2 *C*). During the same period, there was an increase in the calcifications around the left elbow, without pain and without rebound of inflammation (Fig. 1 *B*). Canakinumab therapy is planned to be continued at lower doses.

## Discussion

We report on a case of HFTC, complicated by severe inflammatory flares, which is a previously described complication of the disease.^12^, ^13^, ^14^ The hypothesis of an auto‐inflammatory‐like syndrome is suggested by the clinical and biological presentation, its tendency to evolve through inflammatory flares, and enhanced by the reported presence of macrophages on biopsies of patients with HFTC.^7^ With this rationale, the effect of anti‐IL‐1 treatment has been reported so far only in two cases,^12^ one treated with anakinra, 100 mg subcutaneously daily, then twice daily, and the other one with canakinumab 100 mg every 8 weeks, ie, a sixfold lower dose than the one we used. Both patients had an improvement of general health, a long‐term reduction of CRP, and better action of hypophosphatemic therapies.

In our case, these treatments were relevant in management of inflammatory flares. But the sudden loss of efficacy of the recombinant IL‐1 receptor antagonist anakinra and the partial effect of the anti‐IL‐1 monoclonal antibody canakinumab suggest that the disease is not as IL‐1 dependent as classical auto‐inflammatory syndromes. This suggestion is based on comparison to the clinical effect of anti‐IL‐1 treatments in auto‐inflammatory syndromes observed in our clinical practice; actually, our case remains descriptive, and we did not perform cytokine analyses. To explain the result, the patient could have developed immunization against the drug; we did not measure these antidrug antibodies, but antibodies against anakinra or canakinumab have not been reported to lower efficiency or tolerance of these treatments.^15^, ^16^, ^17^, ^18^, ^19^, ^20^, ^21^, ^22^, ^23^ We cannot exclude that anti‐IL‐1 therapy could be more effective if initiated earlier in the disease course, before a long‐lasting history of diffuse, recurrent tumoral calcinosis.

Hence, anti‐IL‐1 therapies are adjuvant therapies that provide improvement of pain and inflammatory flares of HFTC. They failed in our patient to prevent extension of calcinosis.

## Disclosures

PQ was an investigator of clinical trials with canakinumab, received consulting fees and payment for participation to symposium (<10,000 USD) from Novartis and Swedish Orphan Biovitrum, and received financial support from Novartis and Swedish Orphan Biovitrum to attend congresses. All other authors state that they have no conflicts of interest.
